# Health Status and Disinfection Prior to Grafting Affect the Phenolic Profile of Grapevine Hetero-Grafts and Grafting Yield

**DOI:** 10.3390/plants14030444

**Published:** 2025-02-03

**Authors:** Saša Krošelj, Maja Mikulic-Petkovsek, Domen Kjuder, Anja Pavlin, Matevž Likar, Andreja Škvarč, Katerina Biniari, Denis Rusjan

**Affiliations:** 1 Department of Agronomy, Biotechnical Faculty, University of Ljubljana, Jamnikarjeva 101, SI-1000 Ljubljana, Slovenia; maja.mikulic-petkovsek@bf.uni-lj.si (M.M.-P.); denis.rusjan@bf.uni-lj.si (D.R.); 2 Department of Biology, Biotechnical Faculty, University of Ljubljana, Večna pot 111, SI-1000 Ljubljana, Slovenia; matevz.likar@bf.uni-lj.si; 3 Chamber of Agriculture and Forestry of Slovenia, Agriculture and Forestry Institute Nova Gorica, SI-5101 Nova Gorica, Slovenia; andreja.skvarc@go.kgzs.si; 4 Laboratory of Viticulture, Agricultural University of Athens, Iera Odos 75, 11855 Athens, Greece; kbiniari@aua.gr

**Keywords:** grapevine trunk diseases, Esca, nursery, secondary metabolites, flavanols, stilbenes

## Abstract

Grapevine trunk disease (GTD) is a major threat to grapevine propagation, severely affecting the growth and development of young vines. As one of the most destructive plant diseases in the world, GTD spreads easily through propagation material and threatens the sustainability of vineyards. While effective, biologically friendly treatments remain unavailable. This study investigated the graft yield, the growth potential of grapevine hetero-grafts, and phenolic responses focusing on (i) GTD scion health status (healthy—HLT; asymptomatic—ASYM; symptomatic—SYM) and (ii) disinfection methods. Grafting with HLT scions achieved the highest yield rates, particularly with Serenade^®^ ASO (75%) and BioAction ES (79%), while infected scions showed lower yields. The growth potential of the scions was not affected by the disinfection method or the health status of the scions. Phenolic composition varied between scions, graft callus, rootstock canes, and roots, with scion health status strongly influencing most metabolites. Higher levels of flavanols were observed in HLT scions treated with BioAction ES and Serenade^®^ ASO, with these treatments resulting in 1.6 and 1.5 times higher procyanidin dimer levels, respectively, compared to Beltanol. Flavanols and stilbenes were lower in the callus tissue of grafts with healthy scions compared to infected scions. Rootstock also showed higher levels of catechin and procyanidin dimers in grafts with HLT scions. These results indicate that the health status of scion GTD and the disinfection methods significantly influence the graft yield and phenolic composition, providing valuable insights for GTD management.

## 1. Introduction

Grapevine trunk diseases (GTDs) rank among the most destructive and challenging threats in viticulture in the last 30 years, causing significant yield losses and a marked reduction in grapevine quality [1]. Currently, no effective curative alternative has been introduced, and, consequently, an increase in GTD symptoms has been noted, particularly in nurseries and younger vineyards [2].

Esca, previously also known as “apoplexy”, was among first reported GTDs in many European countries. It is caused by different fungi, among them *Phaeomoniella chlamydospora* P. W. Crous in W. Gams, *Phaeoacremonium aleophilum* W. Gams, Crous, M. J. Wingf. and Mugnai, *Phaeoacremonium minimum* (Tul. and C. Tul.) D. Gramaje, L. Mostert and Crous, and *Fomitiporia mediterranea* Fisher M. [3,4], which are localized in the woody tissues of perennial organs and also in lignified 1-year-old canes [5,6].

Nurseries in Europe are following a certification scheme provided by the European and Mediterranean Plant Protection Organization (EPPO), which dictates testing for certain viruses and phytoplasmas and also checks for the presence of some fungi, including *P. aleophilum, F. punctata,* and *P. chlamydospora.* [7]. Nevertheless, due to the GTD asymptomatic status of mother vines and the large number of wounds that occur during the propagation process, the infections occur in nurseries on a large scale [8,9,10].

Sodium arsenite proved to be very effective in controlling GTD, especially Esca disease [6,11], but it has been banned in the European Union since 2003 [12] due to harmful effects to human health [13]. Despite testing various preparations, active substances, and biological control agents, according to detailed reviews by Gramaje et al. [14] and Mondello et al. [10], no comparable effective treatment is now available to control GTDs [6,9]. In nurseries, mainly preventive measures, such as hot water treatment (HWT) and antagonist and chemical treatments of scions, are used for Esca control [15]. The main reason for the inefficiency of chemicals against GTD-causing agents is linked to difficult access to xylem vessels, where GTD pathogens are located [5,16]. Among chemical preventive fungicides, Chinosol (hydroxyquinoline sulfate) is the most commonly used [15], which is not effective against *Pl. chlamydospora* and *P. minimum* or gives inconsistent results [17,18].

Synthesis of diverse secondary metabolites, such as phenolic compounds, is one of grapevine’s defense mechanisms against pathogen infection [19,20], which exhibits antimicrobial and antioxidant properties that help plants to evade pathogenic infections or protect from toxic effects of reactive oxygen species [19]. One of the first studies evaluating the response of woody parts of the grapevine to infection with the causative agents of white and brown rot was published by Schultz et al. [21], who reported an increased content of stilbenes in infected grapevine wood. This has recently been greatly upgraded by Rusjan et al. [20], who reported that phenolic content in vine wood differed due to Esca infection, wood conditions, and the part of the vine. In all of the studied woods infected by Esca-associated fungi, the accumulation of gallic acid, flavanols, stilbenes, and total analyzed phenolics was clearly observed. Others have also observed a connection between stilbenes, especially *trans*-resveratrol and *ε*-viniferin, and the plant’s response to GTD infection [22,23].

This article aims to assess the impact of various biologically sound disinfectants, alone or combined with hot water treatment (HWT), on the production of vine hetero-grafts using scions with different GTD health statuses. The study evaluates grafting yield, key morphological parameters (number, diameter, and mass of cane and roots), and graft responses at the phenylpropanoid pathway level.

## 2. Results

### 2.1. Grafting Yield (%) and Grafts’ Growth Potential

**Figure 1:**
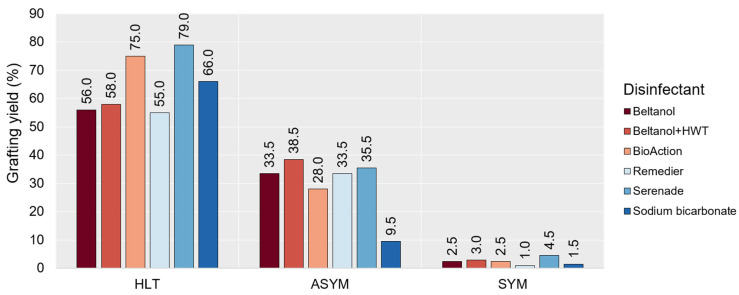
Grafting yield (%; *n* = 200) when grafting with healthy (HLT), asymptomatic (ASYM), and symptomatic (SYM) scions previously disinfected with different disinfectants and one with a combination with heat water treatment (HWT).

Figure 1 represents the grafting yield (%) according to the scions’ GTD health status (HLT, ASYM, SYM) and the disinfection method. After graft ranking, grafting with HLT scions had the highest yield (on average, 64.8%), especially if scions were treated with Serenade^®^ ASO (79.0%) and BioAction ES (75.9%). The average grafting yield with ASYM scions, regardless of disinfection, was 29.7%. With the exception of sodium bicarbonate (9.5%), the grafting yield among other disinfectants was similar, from 29.0% (BioAction ES) to 38.5% (Beltanol + HWT). The significantly lowest grafting yield was achieved by grafting with SYM scions (2.5%). Disinfection of scions with Serenade^®^ ASO deviates from the average with 4.5%.

The growth potential of first-quality vine grafts grafted with previously disinfected scions of different GTD health statuses (HLT, ASYM, SYM) was evaluated by counting the number of canes or root per individual graft and measurements of mass (g) and diameter (mm) of canes and roots. None of the measured parameters were affected by the use of different disinfectants or HWT, and the GTD health status of the scions also had no impact (*α* = 0.05) on the measured parameters (Table S1).

Regardless of the health status and the disinfectant method used, the grafts had between one and six canes per graft, with an average mass of 6.25 ± 0.96 g and an average diameter of 8.11 ± 0.68 mm. They had 4 to 15 roots per graft, with an average mass of 5.41 ± 0.63 g and a diameter of 1.92 ± 0.13 mm (Table S1).

### 2.2. Phenolic Composition of Vine Hetero-Grafts

**Figure 2:**
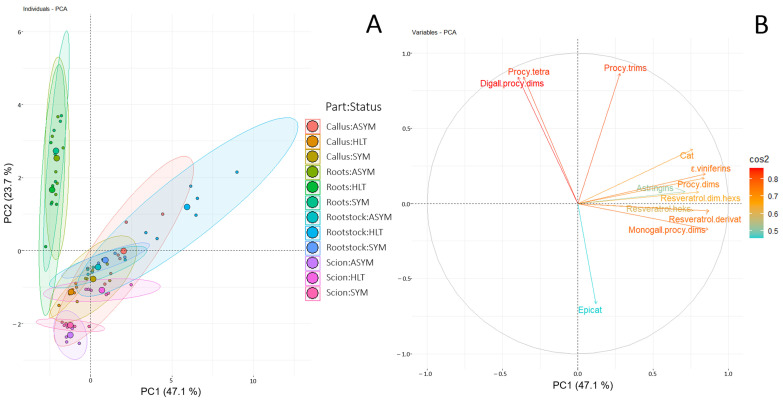
Principal component analysis (PCA) of metabolite content from different vine graft parts (scion, callus, rootstock cane, roots), categorized by different health statuses (healthy: HLT; asymptomatic: ASYM; and symptomatic: SYM), disinfected prior grafting using various disinfectant methods and after graft ranking of the analyzed phenolic compounds (*n* = 4). (**A**): PCA score plots showing the different individuals, colored by part (scion, callus, rootstock canes, roots) and status (HLT, ASYM, SYM) combination. (**B**): PCA loading plot of the 12 most contributing metabolites. The color and the size of the arrows indicate the contribution strength of each metabolite. Abbreviations: Procy: procyanidin; dims: dimers; trims: trimers; tetra: tetramers; monogall: monogalloyl; digall: digalloyl; hexs: hexosides; dim: dimer; cat: catechin.

**Figure 3:**
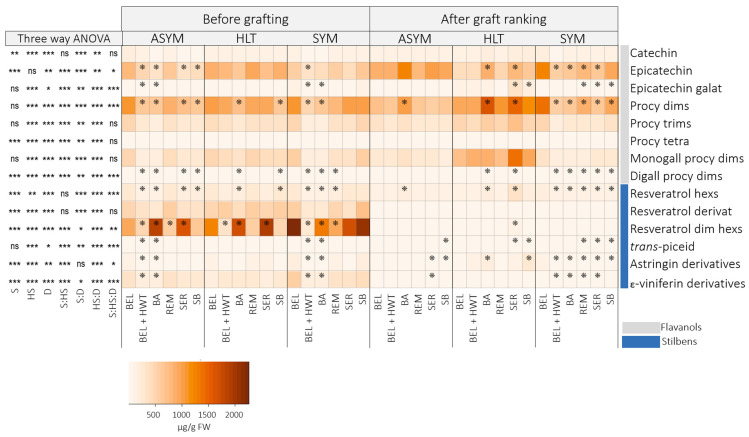
Heatmap illustrating the average contents (µg/g FW) of phenolic compounds in scions categorized by different health statuses (healthy: HLT; asymptomatic: ASYM; and symptomatic: SYM) disinfected prior to grafting using various disinfectant methods (Beltanol: BEL; Beltanol + hot water treatment: BEL + HWT; BioAction ES: BA; Remedier: REM; Serenade^®^ ASO: SER; sodium bicarbonate: SB), sampled before grafting and after graft ranking. On the left side, the results of three-factor ANOVA are presented. S: sampling; HS: health status; D: disinfectant method; Signif. codes: “***”: *p* < 0.001; “**”: *p* < 0.01; “*”: *p* < 0.05; ns: not significant. Stars in the heatmap indicate significant comparisons between the content of phenolics in scions treated with Beltanol and those treated with other disinfectant methods across health status and sampling, obtained through three-way contrasts. Abbreviations: Procy: procyanidin; dims: dimers; trims: trimers; tetra: tetramers; monogall: monoalloyl; digall: digalloyl; hexs: hexosides; dim: dimer.

**Figure 4:**
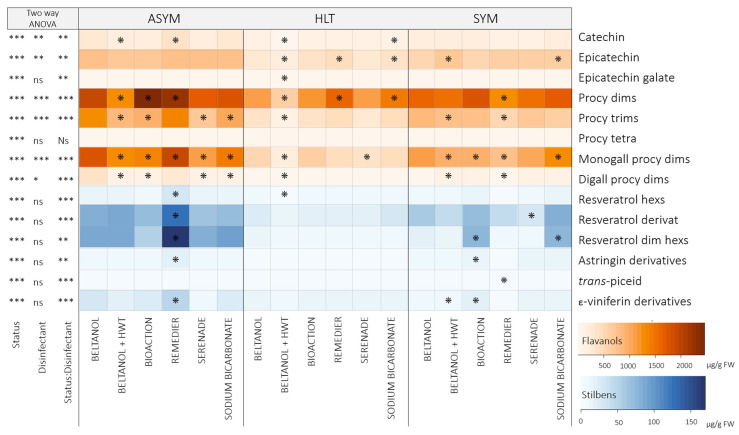
Heatmap depicting the average contents (µg/g FW) of phenolic compounds in callus from grafts initially grafted with scions of varying health statuses (healthy: HLT; asymptomatic: ASYM; and symptomatic: SYM) and disinfected before grafting using different disinfectants (Beltanol, Beltanol + hot water treatment (HWT), BioAction ES, Remedier, Serenade^®^ ASO, and sodium bicarbonate). Samples were collected after graft ranking. The results of a two-factor ANOVA are presented on the left side. Significance levels are denoted as follows: “***” for *p* < 0.001, “**” for *p* < 0.01, “*” for *p* < 0.05, and “ns” for not significant. Stars within the heatmap indicate significant comparisons between the phenolic content in the callus from grafts with scions treated with Beltanol and those treated with other disinfectant methods across different health statuses, as determined through two-way contrasts. Orange tones represent the profile of flavanols, while blue tones represent the profile of stilbenes. Abbreviations: procy: procyanidin; dims: dimers; trims: trimers; tetra: tetramers; monogall: monogalloyl; digall: dialloyl; hexs: hexosides; dim: dimer.

**Figure 5:**
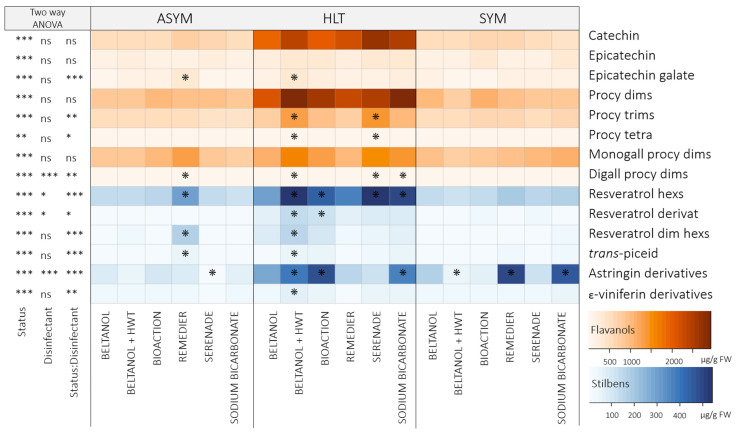
Heatmap depicting the average contents (µg/g FW) of phenolic compounds in rootstock canes from grafts initially grafted with scions of varying health statuses (healthy: HLT; asymptomatic: ASYM; and symptomatic: SYM) and disinfected before grafting using different disinfectants (Beltanol, Beltanol + hot water treatment (HWT), BioAction ES, Remedier, Serenade^®^ ASO, and sodium bicarbonate). Samples were collected after graft ranking. The results of a two-factor ANOVA are presented on the left side. Significance levels are denoted as follows: “***” for *p* < 0.001, “**” for *p* < 0.01, “*” for *p* < 0.05, and “ns” for not significant. Stars within the heatmap indicate significant comparisons between the phenolic content in rootstock canes from grafts with scions treated with Beltanol and those treated with other disinfectant methods across different health statuses, as determined through two-way contrasts. Orange tones represent the profile of flavanols, while blue tones represent the profile of stilbenes. Abbreviations: Procy: procyanidin; dims: dimers; trims: trimers; tetra: tetramers; monogall: monoalloyl; digall: digalloyl; hexs: hexosides; dim: dimer.

**Figure 6:**
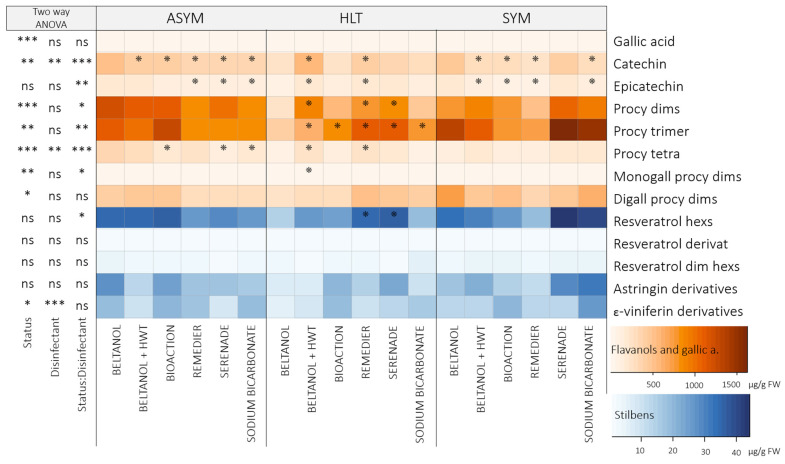
Heatmap depicting the average contents (µg/g FW) of phenolic compounds in roots from grafts initially grafted with scions of varying health statuses (healthy: HLT; asymptomatic: ASYM; and symptomatic: SYM) and disinfected before grafting using different disinfectants (Beltanol, Beltanol + hot water treatment (HWT), BioAction ES, Remedier, Serenade^®^ ASO, and sodium bicarbonate). Samples were collected after graft ranking. The results of a two-factor ANOVA are presented on the left side. Significance levels are denoted as follows: “***” for *p* < 0.001, “**” for *p* < 0.01, “*” for *p* < 0.05, and “ns” for not significant. Stars within the heatmap indicate significant comparisons between the phenolic content in roots from grafts with scions treated with Beltanol and those treated with other disinfectant methods across different health statuses, as determined through two-way contrasts. Orange tones represent the profile of flavanols and hydroxybenzoic acid gallic acid, while blue tones represent the profile of stilbenes. Abbreviations: procy: procyanidin; dims: dimers; trims: trimers; tetra: tetramers; monogall: monogalloyl; digall: dialloyl; hexs: hexosides; dim: dimer.

To characterize the phenolic profiles of vine grafts obtained from scions with different GTD health statuses and that were previously disinfected, 34 individual phenolic compounds were identified in the scions, graft callus, rootstock canes, and roots, with individual variations observed across different tissues, expressed in µg/g of fresh weight (FW). All identified metabolites in each vine graft part were grouped according to chemical properties and were from three major phenolic groups—hydroxybenzoic acids, flavanols, and stilbenes. Gallic acid, the only hydroxybenzoic acid representative identified, was quantified solely in roots, while in the above-ground sections, it was only detected in trace amounts. *Trans*-piceid and epicatechin gallate were exclusively found in the aerial portions of the vine grafts (Table S2; Figure 2, Figure 3, Figure 4, Figure 5 and Figure 6). Scion disinfection prior to grafting and the varying health statuses of the scions did not alter the composition of vine graft parts themselves; however, variations in the levels of individual metabolites were observed.

To obtain a comprehensive picture of the phenolic profiles across various parts of vine grafts, principal component analysis (PCA; Figure 2) was applied, with the first and second components of the PCA model accounting for 47.1% and 23.7% of the total variance, respectively. Looking at the full data, the samples clearly separate along PC1 according to the graft part studied. In particular, the roots of grafts differ considerably in the phenolic profile compared to the upper parts of grafts (Figure 2A). Phenolic compounds that distinguished roots from the upper parts of vine grafts were primarily located on the negative side of PC1 and strongly correlated with diallylated procyanidin dimers and procyanidin tetramers. Additionally, a separation was indicated based on the previous GTD health status of the scions. Specifically, the metabolite content in infected (ASYM and SYM) scions was lower, except for epicatechin. The phenolic profile of rootstock cane and the graft callus was similar, except that rootstock cane grafted with HLT scions and the callus of grafts with ASYM scions were associated with elevated content of stilbenes, catechin, procyanidin dimers, and diallylated procyanidin dimers.

#### 2.2.1. Scions

Phenolic compounds in scions were assessed twice—before grafting and after graft ranking—to examine whether grafting, under studied conditions, induces alterations in the phenolic composition of scions. In both samplings, 15 flavanols and 13 stilbenes were quantified (Figure 3; Table S2). The total flavanol content in the scions exhibited no significant difference after graft ranking compared to before grafting (*p* > 0.05; Figure S1B). Conversely to flavanols, a notable decrease (*p* < 0.001) in the content of total stilbenes and most of the individual representatives (except *trans*-piceid) was noted after grafting (Figure 3 and Figure S1C). On average, HLT scions treated with Beltanol contained, after graft ranking, 9.1-fold, ASYM scions, 9.9-fold, and SYM scions, 8.1-fold lower content of stilbenes compared to before grafting (Figure 3 and Figure S1C).

On the other hand, health status strongly influenced (*p* < 0.01) all analyzed phenolic compounds with the exception of epicatechin. A clear difference in scions after graft ranking was revealed between the content of some individual flavanol representatives, such as procyanidin dimers, monogalloyl procyanidin dimers, and procyanidin trimers, in HLT scions compared to infected grafts (Figure 3). Remarkably, the highest content of these metabolites was consistently observed when scions were previously disinfected with BioAction ES and Serenade^®^ ASO. In the case of procyanidin dimers, previously BioActon-treated HLT scions contained, on average, 1.6-fold (CL: 1.2–2.2), and Serenade^®^-ASO-treated HLT scions contained, on average, 1.5-fold (CL: 1.1–2.1) higher content compared to Beltanol-treated scions (Figure 3).

**Figure 7:**
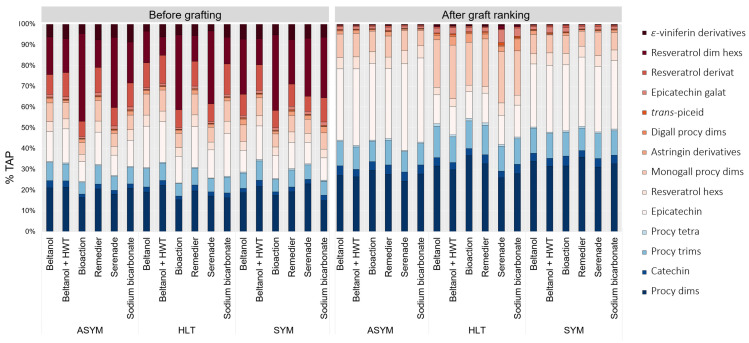
Proportion of phenolic compounds (% of total analyzed phenols; TAP) in scions with different health statuses (healthy: HLT; asymptomatic: ASYM; and symptomatic: SYM) pretreated with various disinfectants and one with hot water treatment (HWT, Beltanol, Beltanol + HWT, BioAction ES, Remedier, Serenade^®^, ASO and sodium bicarbonate) before and after grafting. Abbreviations: dim: dimer; hexs: hexosides; digall: digalloyl; procy: procyanidin; dims: dimers; monogall: monogalloyl; tetra: tetramers; trims: trimers.

Among stilbenes, resveratrol dimer hexosides were, before grafting, the most abundant stilbenes, ranging from 8.8 to 36.1% TAP in HLT scions, 13.4 to 40.3% TAP in ASYM scions, and 12.6 to 36.4% TAP in SYM scions (Figure 7); the proportion was always the highest when scions were treated with BioAction ES (HLT: 36.1 ± 4.5; ASYM: 40.3 ± 3.6; SYM: 36.4 ± 3.5% TAP). The proportions of all stilbenes decreased greatly after grafting. Conversely, the proportions of some flavanols increased after grafting, such as procyanidin dimers, monogalloyl procyanidin dimers, and epicatechin. Notably, the difference in proportions between monogalloyl procyanidin dimers and epicatechin was evident after graft ranking among different health statuses. Regardless of the method of disinfection used, the proportions of epicatechin in infected scions (ASYM: 33.7–40.6%; SYM: 30.4–33.0% TAP) was significantly higher than in HLT scions (13.4–15.4% TAP). On the other hand, the proportion of monogalloyl procyanidin dimers was higher in the HLT scion (20.7% TAP) than in the ASYM (7.4–11.3% TAP) and SYM (7.3–11.3% TAP) scions.

#### 2.2.2. Graft Callus

In the graft callus, 15 flavanols (94.6–98.3% TAP) and 14 stilbenes (1.7–5.4% TAP) were quantified after graft ranking (Figure 4; Table S2), which were strongly influenced by health status (*p* < 0.001). The total flavanol content (Figure S2A) in the callus of grafts with HLT scions was, on average, 3200 ug/g FW (CL: 2882–3519 ug/g FW) lower compared to grafts with ASYM scions and on average 1725 ug/g FW (CL: 2043–1406 ug/g FW) lower compared to grafts with SYM scions. Similar results were obtained for the total stilbene content (Figure S2B), where the callus of grafts with ASYM scions contained, on average, 1.4-fold (CL: 1.1–1.6) more total stilbenes compared to grafts with HLT scions and 0.8-fold (CL: 0.5–1.0) greater content compared to grafts with SYM scions.

All individual phenolics were strongly influenced by health status (*p* < 0.001), while the two-way ANOVA for disinfectants showed significant results only for some flavanol representatives (Figure 4). Procyanidin dimers were the most abundant flavanols in the graft callus (ASYM: 27.2–39.9% TAP; HLT: 42.8–48.5% TAP: SYM: 34.4–36.3% TAP), where the interaction between health status and disinfectants was significant (*p* < 0.001; Figure 4). Compared to the procyanidin dimer content in the callus of grafts with previously Beltanol-treated ASYM scions (1964.60 ± 92.4 µg/g FW; Figure 4), the content in the callus of grafts with BioAction-ES-treated ASYM scions was increased by an average of 489.8 ug/g FW, and that of Remedier-treated ASYM scions was increased by an average of 267.3 ug/g FW. Similar results when ASYM scions were treated with Remedier were also obtained for catechin (1.2-fold higher), monogalloyl procyanidin dimers (1.1-fold higher), and some stilbenes—resveratrol hexosides (2.6-fold higher), resveratrol derivatives (1.6-fold higher), resveratrol dimer hexosides(1.9-fold higher), astringin derivatives (2.4-fold higher), and ε-viniferin derivatives (1.6-fold higher)—when compared to the callus of grafts with Beltanol-treated ASYM scions. However, some individual flavanols were negatively affected by HWT treatment (*p* < 0.05), which was particularly evident in grafts with ASYM and HLT scions (Figure 4). Catechin (ASYM: 1.3-fold lower; HLT: 1.4-fold lower), procyanidin dimers (ASYM: 1.5-fold lower; HLT: 1.7-fold lower), procyanidin trimers (ASYM: 1.4-fold lower; HLT: 2.2-fold lower), and monogalloyl procyanidin dimers (ASYM: 1.4-fold lower; HLT: 2.8-fold lower) were the most negatively affected by HWT.

#### 2.2.3. Rootstock Cane

After graft ranking, the rootstock canes were sampled to obtain a detailed phenolic profile of 15 flavanols and 14 stilbenes (Figure 5; Table S2). The previous GTD health status of scions significantly influenced the content of all metabolites (*p* < 0.01) analyzed in rootstock canes, while disinfection methods influenced only the digalloyl procyanidin content (*p* < 0.05) among flavanols, the total stilbenes content (Figure S3C; *p* < 0.05), and some individual stilbenes (Figure 5).

In particular, differences in the contents of catechin, procyanidin dimers, monogalloyl procyanidin dimers, and resveratrol hexosides in rootstock canes were observed among grafts with scions of varying health statuses. On average, the catechin content was 1.4-fold higher in rootstock canes from grafts with HLT scions compared to grafts with infected scions (CL; ASYM: 1.2–1.5; SYM: 1.3–1.6). Procyanidin dimers were in rootstock canes from grafts with HLT scion content that was, on average, 1665 (CL: 1291–2040) µg/g FW higher than in grafts with ASYM scions and, on average, 1642.4 (CL: 1268–2017) µg/g FW higher than in grafts with SYM scions. Similarly, the content of monogalloyl procyanidin dimer was also, on average, 6.8-fold (CL; ASYM and SYM: 6.7–6.9) higher in rootstock canes from grafts with HLT scions compared to grafts with infected scions.

Previous HWT disinfection of HLT scions significantly influenced the contents of almost all metabolites in rootstock canes after grafting (*p* <0.05) when compared to positive controls. Exceptions were flavanols, including catechin, epicatechin, epicatechin gallate, and monogalloyl procyanidin dimers. In comparison to grafts with HLT-Beltanol-treated scions, grafts with HWT-treated HLT scions had in rootstock canes, on average, 1.6-fold (CL: 1.2–2.1) higher content of total flavanol (Figure S3B) and, on average, 2.0-fold (CL: 1.2–3.6) higher content of total stilbenes (Figure S3C).

#### 2.2.4. Roots

In roots of grapevine grafts, one hydroxybenzoic acid, 15 flavanols, and nine stilbenes were quantified after graft ranking (Figure 6; Table S2). With the exception of *ε*-viniferin derivatives, the previous GTD health status of scions and the previous scion disinfection method did not affect the content of stilbenes (*p* > 0.05). The content of ε-viniferin derivatives in roots from grafts with HLT scions was 12.0 ± 1.1 µg/g FW. In contrast, the content in roots from grafts with infected scions was, on average, lower, at 3.9 µg/g FW (ASYM) and 4.3 µg/g FW (SYM). Similarly, total stilbenes were, on average, 18.9 µg/g FW lower in roots from grafts with HLT scions compared with SYM scions. In comparison to roots from grafts with Beltanol-treated scions, the content of ε-viniferin derivatives was in roots from grafts with HWT-treated scions, on average, 7.4 µg/g FW higher with HWT and 7.6 µg/g FW higher with sodium-bicarbonate-treated scions.

The content of gallic acid (Figure 6), the only quantified hydroxybenzoic representative, was in roots of grafts with HLT scions 17.1 ± 1.1 µg/g FW, and it was significantly lower when compared to roots from grafts with ASYM (on average, 4.9 µg/g FW higher) and SYM (on average, 5.6 µg/g FW higher).

Procyanidins were the most abundant flavanols in graft roots, which were significantly affected by the health status of grafted scions (*p* < 0.05). Synthesis in roots from grafts with infected scions was especially enhanced (Figure 6). Also, total flavanols (Figure S4B) and individual representatives (Figure 6) showed similar behavior, with the exception of epicatechin. The average content of total flavanols in roots from grafts with HLT scions was 2524 ± 186 µg/g FW; on the other hand, in roots from grafts with infected scions, the content of flavanols was, on average, 27.5% (ASYM) and 21.5% (SYM) higher. In roots from grafts with HLT scions treated with HWT, individual flavanols, with the exception of digalloyl procyanidin dimers, increased compared to Beltanol-treated scions. Similarly, this was also the case when scions were treated with Remedier.

### 2.3. Correlation Between Phenolics and Grafting Yield

**Figure 8:**
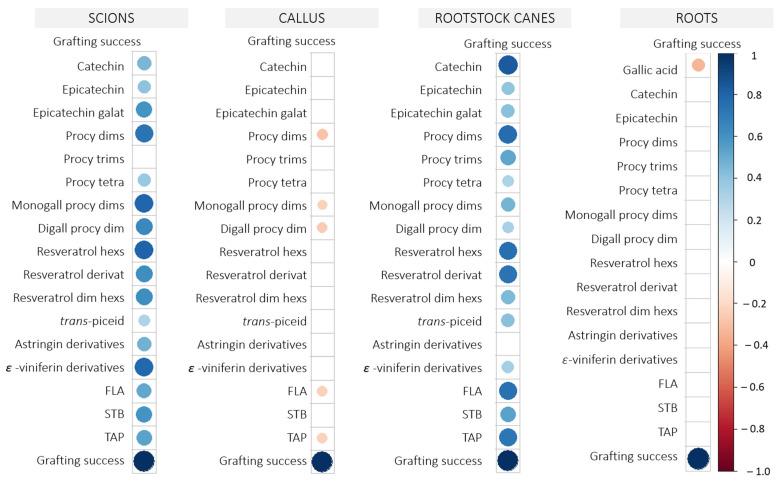
The Pearson correlation coefficient between the percentage of grafting success (*n* = 200) and the phenolic compound content (*n* = 4) from different vine graft parts (scion, callus, rootstock cane, roots), regardless of health status and disinfection method. Positive correlations are colored in blue, and negative correlations are in red. The size and color intensities represent the correlation level. Abbreviations: dim: dimer; hexs: hexosides; digall: digalloyl; procy: procyanidin; dims: dimers; monogall: monogalloyl; tetra: tetramers; trims: trimers.

The correlation matrices were constructed using the entire dataset, irrespective of health status and the employed scion disinfection method. They were generated independently for scions, rootstock canes, and roots to examine potential correlations between metabolite contents and grafting yield (Figure 8). The most significant positive correlations were found in scions and rootstock canes. In vine graft roots, only gallic acid showed a significant negative correlation with grafting yield (r^2^ = −0.34, *p* < 0.01), and in the graft callus, individual phenolics—procyanidin dimers (r^2^ = −0.29, *p* < 0.05), monogalloyl procyanidin dimers (r^2^ = −0.23, *p* < 0.05), and digalloyl procyanidin dimers (r^2^ = −0.28, *p* < 0.05)—had a weak negative correlation with grafting yield.

In scions, grafting yield was positively correlated with mostly all phenolic compounds (Figure 8). The strongest correlations were with procyanidin dimers (r^2^ = 0.74, *p* > 0.001), monoallylated procyanidin dimers (r^2^ = 0.79, *p* < 0.001), resveratrol hexosides (r^2^ = 0.81, *p* < 0.001), and *ε*-viniferin derivatives (r^2^ = 0.79, *p* < 0.001).

Similarly, grafting yield in rootstock canes was also stringently correlated (Figure 8) with catechin (r^2^ = 0.83, *p* < 0.001), procyanidin dimers (r^2^ = 0.77, *p* < 0.001), resveratrol hexosides (r^2^ = 0.76, *p* < 0.001), and resveratrol derivate (r^2^ = 0.73, *p* < 0.001).

## 3. Discussion

This study aimed to evaluate the impacts of various disinfectants and one combined with hot water treatment (HWT) on the production of vine hetero-grafts using scions with different GTD health statuses (healthy: HLT; asymptomatic: ASYM; symptomatic: SYM). Additionally, it was examined how the phenolic profiles of the grafts varied across different parts (scion, rootstock cane, callus, and roots) in response to different disinfection methods and scion health statuses.

The results demonstrated varying levels of grafting yield among different scion GTD health statuses and disinfection methods (Figure 1). Grafting with HLT scions exhibited the highest yield rates, particularly when treated with Serenade^®^ ASO, a preventive fungicide comprising the bacterium *Bacillus amyloliquefaciens* (former *B. subtilis*) strain QST 713, which is recognized as a microbial disrupter of pathogen cell membranes and capable of forming endospores and producing various antibiotics; also, it may induce plant-mediated resistance in host plants [24]. High rates were also obtained using BioAction ES, a foliar fertilizer comprising extracts of clove, lemon juice, garlic oil, and peppermint (3%), along with copper (4.5%) and MicroSap^®^ microcrystals (15%), which, according to the manufacturer, can bind to natural substances, penetrate through the trunks, and reach conductive tissues, thus enhancing the plant’s immune system and fostering natural resistance to diseases [25]. This suggests that certain disinfectants can effectively mitigate the risk of graft failure associated with diseases, especially when applied to healthy GTD-free scions. However, it is noteworthy that the effectiveness of disinfectants varied depending on scions’ health status regarding GTD. For instance, sodium bicarbonate, which previously showed antifungal effects on a wide range of pathogenic fungi, such as the *Botrytis cinerea* on table grapes (Gabler and Smilanick, 2001), *Ulocladium chartarum*, *Aspergillus niger*, *Fusarium semitectum*, and *Geotrichum candidum* [26], in this case showed poor performance for infected scions. This indicates the limited efficacy of sodium bicarbonate against Esca-associated pathogens, particularly *Alternaria* sp., as, in the same asymptomatic vines, regarding Esca, from which ASYM grafts were also obtained, Rusjan et al. [20] reported only these fungi in all wood states (healthy, necrotic, and rotted) of asymptomatic vines. Also, grafting with SYM scions yielded the lowest success rates, regardless of the disinfection method used. In the same symptomatic vines from which SYM scions were cut, Botryosphaeriaceae sp. was found in healthy wood, while *Phaeomoniella chlamydospora* and *Fomitiporia mediterranea* were detected in rotted or necrotic wood of symptomatic vines [20]. This suggests that all disinfectants are poorly effective in grafting with SYM scions against the aforementioned Esca-associated fungi.

Interestingly, the growth potential of the first-quality grafts (Table S3), measured using parameters like the number, mass, and diameter of canes and roots, remained unaffected by both the disinfection methods and the health status of the scions.

The phenolic response of woody parts of grapevine grafts to grafting with scions of varying health statuses in relation to GTD and prior disinfection with different disinfectants, including one in combination with HWT, was investigated. Synthesis of phenolic compounds is one of the grapevine’s defense mechanisms against pathogen infection [19,20]. In particular, enhanced synthesis of gallic acid, *trans*-resveratrol, and *ε*-viniferin is associated with grapevine’s response to GTD [22,23]. Also, grafting and wounding stimulate defense mechanisms and local secondary synthesis of secondary metabolites, which have been scrutinized, especially in graft incompatibility studies [27,28]. These studies mainly highlight processes at the graft callus, as phenolic compounds play important roles in various developmental and differentiation processes during callus formation [29], while other parts of grapevine grafts remain less studied in terms of their biochemical response during grafting. The phenolic content of the vine grafts (Figure 2) varied significantly across different parts (scions, rootstock canes, graft callus, and roots) and was mostly influenced by the scion’s GTD health status.

The analysis of phenolic compounds in scions (Figure 3) revealed variations in content before and after grafting. While the total flavanol content remained unchanged, there was a notable decrease in the total stilbenes content and most individual stilbene representatives after grafting. Grafting can trigger a restructuring of metabolism in plants, as energy and resources are diverted to the renewal and growth of new tissues [30]. This can lead to a change in synthesis and the accumulation of metabolites to the graft callus, as was also was revealed in the case of the scion genotype *V. vinifera* cv. Merlot Noir grafted onto SO4 rootstock [28], where an enhanced synthesis of stilbenes *trans*-piceatannol, *trans*-*δ*-viniferin, parthenocisin, *trans*-resveratrol, pallidol, isohopeaephenol, hopeaphenol, and *α*-viniferin was observed. No such changes were observed in flavanols. The opposite was observed in this study, as mainly flavanols—catechin, procyanidin dimers, procyanidin trimer, monogalloyl procyanidin dimers, and digalloyl procyanidin dimers—and two stilbenes—resveratrol derivative and resveratrol dimer hexosides—accumulated strongly at the callus (Figure 4) compared to the phenolic profile of the scions (Figure 3).

The health status of the scions significantly influenced all analyzed metabolites in different upper parts of the vine grafts (scion, callus, rootstock cane; Figure 3, Figure 4 and Figure 5). In general, flavanols and stilbenes were in the callus of grafts with HLT scions in significantly lower amounts compared to grafts with infected scions. On the other hand, scions and rootstock canes from grafts with infected scions exhibited lower content compared to healthy scions, except for the epicatechin content in the scions, which was clearly seen, with a discernible separation, through PCA analysis (Figure 2). This is in contrast to reports by Rusjan et al. [20], Amalfitano et al. [22], and Martin et al. [23], where higher levels of metabolites are reported in infected tissues. The reason could be that the phenylpropanoid synthetic pathway could divert its metabolites incorporated into insoluble lignin and lignin-like polymers in response to infection with the aim of strengthening cell walls and structural rigidity, which prevents their extraction through the standard phenolic extraction procedure [11,31]. Another reason could be the tannin–protein complexes, which are formed when plant tannins bind with fungi-derived enzymes. They are insoluble and cannot be analyzed through the phenolic extraction procedure used [32].

Significant differences were observed in the content of individual flavanol representatives in HLT scions after grafting (Figure 3), particularly in the content of procyanidin dimers, monogalloyl procyanidin dimers, and procyanidin trimers. The highest contents were observed in scions treated with BioAction ES and Serenade^®^ ASO, which is linked to the expression of PAL (Phenylalanine ammonium lyase), a key enzyme of the phenylpropanoid pathway, which is induced following the application of Serenade^®^ ASO [33]. The same authors reported that the preparation also induced BGLU (*β*-glucosidases family genes), which are involved in the formation of lignin and lignin derivatives [34] and could potentially influence graft union formation, as lignin accumulates to act as a physical and antimicrobial barrier at the site of wounds and is necessary for xylem formation [27,30].

The correlation analysis (Figure 8) later revealed significant associations between grafting yield and the aforementioned flavanols in scions—procyanidin dimers and monogalloyl procyanidin dimers. Stilbenes—resveratrol hexosides and ε-viniferin derivatives—were also strongly positive correlated with grafting yield. Similarly, Loupit et al. [28] also found in scions 33 days after grafting a positive correlation between grafting yield and the content of *trans*-piceatannol and *trans*-resveratrol in scions. They also reported negative correlations between some flavanols in the graft callus and the grafting yield, which was also true in the case of this experiment, as procyanidin dimers, monogalloyl procyanidin dimers, and digalloyl procyanidin dimers had a weak negative correlation with the grafting yield.

As mentioned, the response of the graft callus after grafting healthy rootstock canes with infected scions was much different than that of the scions and rootstock canes themselves (Figure 4). In addition, disinfection of ASYM scions with Remedier resulted in an increased content of certain phenolics in the graft callus compared to grafts with Beltanol-treated scions. This could be attributed to the induced graft defense response by the *Trichoderma* species in the Remedier disinfectant, which can trigger the graft’s defense mechanisms, leading to the increased production of phenolic compounds [35].

Rootstock canes exhibited significant differences in phenolic compound contents based on the health status of the grafted scions (Figure 5). In particular, variations were observed in the contents of catechin, procyanidin dimers, monoallylated procyanidin dimers, and resveratrol hexosides among rootstock canes from grafts with healthy and infected scions. Additionally, the HWT of healthy scions significantly influenced the content of most metabolites in rootstock canes after grafting, resulting in higher content compared to Beltanol-treated scions. Interestingly, such a behavior did not appear in the HWT-treated scions (Figure 3), whereas the opposite was true for the graft callus (Figure 4). The cause could be an interaction between the rootstock cane and the HWT-treated scions, which may emit signals or molecules that affect metabolism and defense responses of the rootstock [36]. Rai et al. [37] summarized current knowledge concerning how salicylic acid, a signal molecule that plays an important role in stress situations, senses and triggers a heat stress response in plants. The heat response pathway in plants involves a complex regulatory network of multiple signaling pathways, which also includes post-transcriptional histone/chromatin modifications in RNA-dependent DNA methylation, which play a key role in reprogramming plant phenology to maintain long-term stress memory [37]. Also, salicylic acid can move downward from the scion through the phloem [38] and transmit the stress signal to the rootstock, where it stimulates the PAL enzyme, as in the case of salicylic acid treatment in apples [39]. Salicylic acid’s involvement in rootstock–scion communication was also suggested by Fu et al. [40].

The phenolic composition in roots varied depending on the scion’s GTD health status and the disinfection method (Figure 6). While the health status of scions significantly influenced all analyzed metabolites, disinfection methods affected only the content of digalloyl procyanidin among flavanols and total stilbenes. Notably, significant differences were observed in the content of procyanidins, with infected scions exhibiting enhanced synthesis in the roots. To our knowledge, phenolic compounds in the roots of grapevine grafts have not been determined until now. We can only compare the profiles with older vines, where the absence of phenolic acids and flavonoids is reported [41,42].

## 4. Material and Methodes

### 4.1. Chemicals and Reagents

For surface disinfection of all graft materials prior to phenolic extraction and for phenolic extraction, methanol from Sigma-Aldrich (34860, St. Louis, MO, USA) was used. Formic acid from Fluka Chemie (06561, Buchs, Sankt Gallen, Switzerland) was also used for phenolic extraction. All aqueous solutions were prepared using double-distilled water from the Milli-Q water system (Millipore, Bedford, MA, USA). For the quantification of individual phenolic compounds, the following standards were used: procyanidin B1 (42157), catechin (C1788), epicatechin (45300) (Fluka Chemie, Buchs, Sankt Gallen, Switzerland), gallic acid (G7384), and resveratrol (R5010) (Sigma-Aldrich, St. Louis, MO, USA). For HPLC mobile phases, acetonitrile (34851, Sigma-Aldrich, St. Louis, MO, USA), formic acid (06561, Fluka Chemie, Buchs, Sankt Gallen, Switzerland), and sulfuric acid (339741, Sigma-Aldrich, St. Louis, MO, USA) were used.

### 4.2. Plant Material and Experimental Design

This study focused on scions collected from grapevines (*Vitis vinifera* L.) of the ’Cabernet Sauvignon’ cultivar, grafted on SO4 rootstock (*Vitis berlandieri* × *Vitis riparia*) and planted in a vineyard at Brdice pri Neblem (46.004452° N, 13.509907° E; Goriška Brda, Slovenia). A total of 20 vines were marked, including 5 healthy vines and 15 showing typical symptoms of grapevine trunk disease (GTD) on leaves, canes, or trunks. Symptomatic vines were identified based on the criteria described by Rusjan et al. [18], such as tiger-like chlorotic and necrotic spots on leaves or dead canes and spurs. Healthy vines were selected at a considerable distance from symptomatic vines to minimize the risk of GTD infection. All vines had previously been tested for viruses, including GFLV, ArMV, GFkV, GVA, GLRaV-1, and GLRaV-3, through ELISA and were confirmed to be virus-free. Pathogenic fungi associated with GTD (e.g., Esca, *Eutypa*) were also identified in both symptomatic and asymptomatic vines from which scions were collected.

During winter pruning, canes were cut from the marked vines and immediately bundled and transported to the Selection Grapevine Centre Vrhpolje (Vipava, Slovenia). Healthy canes from virus-free vines were first cut and labeled as healthy scions (HLT). Subsequently, canes from vines affected by GTD were processed and categorized as symptomatic (SYM; with visually detectable necrosis in the vascular tissue on cross-sections) and asymptomatic (ASYM; with no visible necrosis in the vascular tissue on cross-sections).

The scions were stored separately according to their health status (HLT, ASYM, SYM) in transparent PVC bags at 2 °C until grafting. Rootstocks of the highest health and sanitary quality of the variety Kober 5BB (*Vitis berlandieri* × *Vitis riparia*) were provided by the Clonal Selection Centre Ivanjkovci (Ormož, Slovenia). To avoid cross-contamination with fungal infections, these rootstocks were stored in PVC bags at 2 °C in a separate storage chamber. For grafting, 200 scions and 200 rootstocks were selected for each disinfection method listed in Table S3 to ensure representation of all scion health statuses (HLT, ASYM, SYM).

Separately by treatment, scions were soaked for 12 h in different disinfectant preparations, listed with descriptions in Table S3, while all rootstock canes were treated with Beltanol, as it is the only registered agent in Slovenia for graft disinfection. Scions treated with Beltanol + HWT were first treated with HWT at 50 °C for 45 min according to the EPPO standard [7], cooled in lukewarm water (21–22 °C), and then, before grafting, soaked for 12 h in Beltanol. After disinfection, scions were grafted separately according to their health status. Scion and rootstock cane pairs were bench-grafted using the omega graft technique. From there on, the production of vine grafts was arranged as usual in the following order in a grapevine nursery: paraffining, callusing, planting in growing beds, spraying (fungicides against downy mildew and oidium and twice with insecticide against American grapevine leafhopper (*Scaphoideus titanus*), a vector of Flavescence dorée), digging up the dormant grafts, graft ranking, and storing.

### 4.3. Grafting Yield and Graft Evaluation

According to the graft ranking, only first-quality grafts were included in the graft yield evaluation. The grafting yield was calculated according to the following formula:

Graft yield (%) = (number of first-class grafts/total number of grafts; *n* = 200) × 100%.

For the grafting study, 10 random grafts of first-class quality were selected for each treatment according to the graft ranking. On these grafts, all canes were counted, and the diameter (cm) was measured for primary canes growing directly from the scions. The growth potential of each graft was assessed by cutting primary and secondary canes (those growing from the primary cane) close to the scion, shortening them to 5 cm in length, and weighing them (g).

The roots of each first-quality vine graft were also examined. The total number of roots was counted, and their diameter (cm) was measured at 3 cm from the heel of the graft. The roots were then shortened to a length of 10 cm from the graft heel and weighed (g).

### 4.4. Phenolic Compound Analysis

Sampling of first-quality grapevine grafts for phenolic analysis using high-performance liquid chromatography coupled with mass spectrometry (HPLC/MS) was conducted following graft ranking. Scions were also sampled prior to grafting. At each sampling date, 15 grafts (or scions) per treatment were collected and stored in PVC bags at 4 °C for subsequent analysis.

The extraction of phenolic compounds from scions, the graft callus, rootstock canes, and roots was performed following the protocol described by Rusjan et al. [20]. Each of the five grafts per treatment was first sterilized with 80% ethanol and then divided into individual parts: roots, the scion, the graft callus, and the rootstock cane. The wood from these sections was finely powdered using liquid nitrogen.

Approximately 0.2 g of each sample was weighed and extracted with 1.7 mL of a solution containing 3% formic acid in 80% methanol. The extraction process lasted for 1 h at 0 °C in an ultrasonic bath (Sonis 3, Iskra PIO, Šentjernej, Slovenia). Afterward, the samples were centrifuged at 10,000 rpm for 7 min with Eppendorf Centrifuge (5810 R, Germany). The resulting supernatant was filtered through a Chromafil AO-20/25 polyamide filter (Macherey-Nagel, Düren, Germany), transferred to vials, and stored at −80 °C until analysis through HPLC.

Quantification of individual phenolic compounds was performed as reported by Gacnik et al. [37] using an Accela HPLC system (Thermo Scientific, San Jose, CA, USA) equipped with a diode array detector (DAD). Detector wavelengths were set at 280 nm for flavanols and hydroxybenzoic acids and 350 nm for stilbenes to target specific phenolic groups in the plant material. Detailed HPLC conditions and equipment specifications are given in Table S4. Identification of phenolic compounds was achieved through comparison of retention times with known standards and through mass spectrometry (LTQ XL™ Linear Ion Trap Mass Spectrometer; Thermo Scientific) using electrospray ionization. Mass spectrometry conditions are also outlined in Table S4, following the protocol reported by Gacnik et al. [39]. Extracts were eluted using a linear gradient, as described in Table S4. Data acquisition and processing were performed using Excalibur 2.2 software (Thermo Fisher Scientific, Waltham, MA, USA). The concentrations of phenolic compounds were calculated from the peak areas of the samples and their corresponding standards, and the results were expressed as µg/g fresh weight (FW). All identified metabolites were grouped according to their chemical characteristics. In addition, total flavanols, total stilbenes, and total analyzed phenolics (TAP) were calculated by summing the respective groups of compounds.

### 4.5. Statistic Analysis

The experiment was designed with multiple factors. The health status of scions included one factor with three levels—HLT: healthy; ASYM: asymptomatic; SYM: symptomatic. The second factor included different disinfectant suspensions or methods with 6 levels (Beltanol, Beltanol + HWT, Serenade^®^ ASO, BioAction ES, Remedier, and sodium bicarbonate). The use of Beltanol as a standard procedure for disinfecting parts of grafts in nurseries was set as a positive control. Another factor, sampling timing (before grafting and after graft ranking), was considered for the analysis of phenolic compounds in scions. Principle component analysis (PCA), heatmap, boxplot, and statistical tests were performed using R-commander statistical software (R Formation for Statistical Computing, Auckland, New Zealand, 2021) using the ggplot2 (version 3.4.1), FactoMineR (version 2.10), multcomp (version 1.4.22), and agricolae (version 1.3.5) packages. Differences among treatments for growth potential parameters and for the phenolic content in roots, the graft callus, and the rootstock cane were determined with two-way ANOVA and for phenolic content in scions with three-way ANOVA. A residual analysis was conducted to test the assumptions of ANOVA. Outliers were evaluated using the box plot method, normality was assessed using Shapiro–Wilk’s normality test, and homogeneity of variances was examined using Levene’s test. If a significant interaction was observed, statistical differences were analyzed using user-defined contrasts with the glht function. All comparisons were made relative to the positive control. If the interaction was not significant, pairwise comparisons between the different health statuses of scions or the disinfection method used were conducted using the emmeans_test() function.

## 5. Conclusions

This study evaluated the efficacy of different disinfectants, including one combined with hot water treatment (HWT), in the production of grapevine hetero-grafts using scions with different GTD health statuses (healthy: HLT; asymptomatic: ASYM; symptomatic: SYM). The results showed that HLT scions treated with Serenade^®^ ASO or BioAction ES achieved the highest grafting success rates, highlighting the potential of these disinfectants to reduce the risk of graft failure, particularly in GTD-free scions. Conversely, symptomatic (SYM) scions consistently showed low grafting success regardless of disinfection, highlighting the limited efficacy of current treatments against advanced infections. Phenolic responses varied between vine parts and were influenced by scion health and disinfection, suggesting a complex interplay between biochemical pathways and disease management strategies. These results provide valuable insights for improving grafting success and reducing economic losses in viticulture and highlight the need for early intervention and targeted treatments in the fight against GTD.

## Data Availability

The data used to support the findings of this study are available from the corresponding author upon request.

## Supplementary Materials

The following supporting information can be downloaded at: https://www.mdpi.com/article/10.3390/plants14030444/s1, Table S1: The growth potential of high-quality vine hetero-grafts grafted with previously disinfected scions using different disinfectants (Beltanol, Beltanol + hot water treatment (HWT), BioAction ES, Remedier, Serenade^®^ ASO, and sodium bicarbonate) and of different health statuses (HLT: healthy; ASYM: asymptomatic; SYM: symptomatic); Table S2: Phenolic compounds identified in the scion, callus, rootstock canes, and roots; Table S3: Methods for disinfection and preparation of disinfectant suspensions for grapevine scions’ disinfection before grafting in 2017; Table S4: HPLC and MS conditions, based on and Gacnik et al. [37]; Figure S1: Content (µg/g FW) of TAP (total analyzed phenolic content) (A), FLA (flavanols) (B), and STB (stilbenes) (C) in scions categorized by different health statuses (healthy: HLT; asymptomatic: ASYM; and symptomatic: SYM) disinfected prior to grafting using various disinfectant methods (Beltanol, Beltanol + hot water treatment (HWT), BioAction ES, Remedier, Serenade^®^ ASO, sodium bicarbonate) and sampled before grafting (I) and after graft ranking (II); Figure S2: Content (µg/g FW) of TAP (total analyzed phenolic content) (A), FLA (flavanols) (B), and STB (stilbenes) (C) in the graft callus categorized by different health statuses (healthy: HLT; asymptomatic: ASYM; and symptomatic: SYM) disinfected prior to grafting using various disinfectant methods (Beltanol, Beltanol + hot water treatment (HWT), BioAction ES, Remedier, Serenade^®^ ASO, sodium bicarbonate) and sampled after graft ranking. Figure S3: Content (µg/g FW) of TAP (total analyzed phenolic content) (A), FLA (flavanols) (B), and STB (stilbenes) (C) in rootstock canes categorized by different health statuses (healthy: HLT; asymptomatic: ASYM; and symptomatic: SYM) disinfected prior to grafting using various disinfectant methods (Beltanol, Beltanol + hot water treatment (HWT), BioAction ES, Remedier, Serenade^®^ ASO, sodium bicarbonate) and sampled after graft ranking; Figure S4: Content (µg/g FW) of TAP (total analyzed phenolic content) (A), FLA (flavanols) (B), and STB (stilbenes) (C) in roots categorized by different health statuses (healthy: HLT; asymptomatic: ASYM; and symptomatic: SYM) disinfected prior to grafting using various disinfectant methods (Beltanol, Beltanol + hot water treatment (HWT), BioAction ES, Remedier, Serenade^®^ ASO, sodium bicarbonate) and sampled after graft ranking; Figure S5: Principal component analysis (PCA) of metabolite content from different vine graft parts (scion, callus, rootstock cane, roots), categorized by different health statuses (healthy: HLT; asymptomatic: ASYM; and symptomatic: SYM), disinfected prior to grafting using various disinfectant methods and after graft ranking the analyzed phenolic compounds (n = 4). PCA score plots showing the different individuals, colored based on the contribution strength of each combination of disinfectant_health and status_graft part.
