# Supplementary material for: Health Status and Disinfection Prior to Grafting Affect the Phenolic Profile of Grapevine Hetero-Grafts and Grafting Yield
Source: Plants (Basel). 2025 Feb 3;14(3):444. doi: 10.3390/plants14030444 (PMC11820403; doi:10.3390/plants14030444)
Supplement: Supplementary file 1 [file plants-14-00444-s001.zip › plants-3444080-supplementary.pdf]

SUPPLEMENTARY MATERIAL

Table S1: The growth potential of 1-st quality vine hetero-grafts, grafted with previously disinfected scions using different disinfectants (Beltanol, Beltanol + Hot Water Treatment (HWT), BioAction ES, Remedier, Serenade® ASO and sodium bicarbonate) of different health statuses (HLT: healthy, ASYM: asymptomatic, SYM: symptomatic).

| Status | Disinfectant | Cane mass (g) | Cane number | Cane diameter (mm) | Root mass (g) | Root number | Root diameter (mm) |
|---|---|---|---|---|---|---|---|
| HLT | Beltanol | 5.57±1.05 | 2.70±0.40 | 8.14±0.83 | 5.30±0.43 | 9.20±0.89 | 1.91±0.15 |
| | Beltanol+HWT | 7.79±1.52 | 2.70±0.42 | 8.15±0.90 | 6.39±0.74 | 9.90±0.85 | 1.96±0.12 |
| | BioAction ES | 4.86±0.51 | 2.20±0.33 | 7.70±0.40 | 4.44±0.54 | 9.00±0.73 | 1.83±0.13 |
| | Remedier | 7.05±0.85 | 2.50±0.40 | 8.11±0.64 | 5.42±0.39 | 10.3±0.68 | 1.93±0.11 |
| | Serenade® ASO | 5.94±0.60 | 2.50±0.34 | 8.88±0.64 | 5.00±0.28 | 9.20±0.73 | 2.07±0.13 |
| | Sodium bicarbonate | 4.87±0.41 | 2.10±0.23 | 7.84±0.44 | 3.97±0.35 | 9.20±0.89 | 1.77±0.09 |
| ASYM | Beltanol | 5.69±1.10 | 2.30±0.26 | 7.51±0.58 | 5.23±0.90 | 7.90±0.71 | 2.01±0.15 |
| | Beltanol+HWT | 5.66±0.43 | 2.60±0.40 | 6.91±0.57 | 4.80±0.38 | 9.80±0.65 | 1.65±0.09 |
| | BioAction ES | 4.84±0.42 | 1.90±0.35 | 6.74±0.30 | 4.08±0.33 | 9.40±0.62 | 1.76±0.12 |
| | Remedier | 7.42±0.52 | 2.70±0.26 | 8.59±0.34 | 6.88±0.43 | 10.6±0.78 | 1.99±0.12 |
| | Serenade® ASO | 7.64±0.99 | 3.00±0.52 | 8.20±0.76 | 6.18±0.68 | 8.40±0.78 | 1.93±0.05 |
| | Sodium bicarbonate | 6.83±1.13 | 2.20±0.39 | 9.88±0.87 | 6.58±0.69 | 8.30±0.52 | 2.14±0.13 |
| SYM | Beltanol | 4.43±1.54 | 2.00±0.32 | 7.32±1.12 | 4.94±1.42 | 8.60±1.63 | 1.87±0.17 |
| | Beltanol+HWT | 5.83±0.25 | 2.80±0.66 | 8.86±1.33 | 4.93±0.74 | 8.40±1.29 | 1.93±0.19 |
| | BioAction ES | 7.75±1.43 | 2.40±0.40 | 9.80±0.82 | 5.02±0.37 | 7.40±1.17 | 1.87±0.24 |
| | Serenade® ASO | 4.29±1.33 | 2.00±0.32 | 7.76±1.03 | 4.48±0.88 | 9.20±1.39 | 1.72±0.11 |
| | Sodium bicarbonate | 5.63±3.18 | 2.33±1.33 | 6.84±1.5 | 6.24±1.87 | 7.00±0.58 | 2.26±0.29 |
| | Status | NS | NS | NS | NS | NS | NS |
| | Disinfectant | NS | NS | NS | NS | NS | NS |
| | S×D | NS | NS | NS | NS | NS | NS |
| | Average | 6.25±0.96 | 2.43±0.36 | 8.11±0.68 | 5.41±0.63 | 9.12±0.77 | 1.92±0.13 |

The results of a two-factor ANOVA are presented with mean value ± standard error. NS – non-significant, $p > 0.05$. The last row shows the average for each measured parameter, regardless of status and disinfectant.

Table S2: Phenolic compounds identified in scion, rootstock cane, callus and roots.

| Phenolic compound | Scion | Rootstock cane | Callus | Roots |
|---|---|---|---|---|
| Gallic acid | NQ | NQ | NQ | + |
| Procyanidin dimer 1 | + | + | + | + |
| Procyanidin dimer 2 | + | + | + | + |
| Procyanidin dimer 3 | + | + | + | + |
| Procyanidin dimer 4 | + | + | + | + |
| Procyanidin dimer 5 | + | + | + | + |
| Catechin | + | + | + | + |
| Procyanidin trimer 1 | + | + | + | NQ |
| Procyanidin trimer 2 | + | + | + | + |
| Procyanidin trimer 3 | NQ | NQ | NQ | + |
| Procyanidin trimer 4 | + | + | + | + |
| Procyanidin trimer 5 | NQ | NQ | NQ | + |
| Procyanidin trimer 6 | NQ | NQ | NQ | + |
| Procyanidin tetramer | + | + | + | + |
| Epicatechin | + | + | + | + |
| Resveratrol he+oside 1 | + | + | + | + |
| Resveratrol he+oside 2 | + | + | + | NQ |
| Resveratrol he+oside 3 | + | + | + | + |
| Resveratrol he+oside 4 | + | + | + | NQ |
| Monogalloyl procyanidin dimer 1 | + | + | + | + |
| Monogalloyl procyanidin dimer 2 | + | + | + | NQ |
| Astringin 1 | + | + | + | + |
| Astringin 2 | + | + | + | NQ |
| Astringin 3 | NQ | NQ | NQ | + |
| Digalloyl procyanidin dimer | + | + | + | + |
| *Trans*-piceid | + | + | + | NQ |
| Epicatechin gallate | + | + | + | NQ |
| Resveratrol derivate | + | + | + | + |
| Resveratrol dimer he+oside 1 | + | + | + | NQ |
| Resveratrol dimer he+oside 2 | + | + | + | + |
| ε - viniferin 1 | + | + | + | + |
| ε - Viniferin 2 | + | + | + | + |
| ε - Viniferin 3 | + | + | + | + |
| *ε* - viniferin 4 | + | + | + | NQ |
| Unidentified 518 | + | + | + | NQ |
| Unidentified 519 | + | + | + | NQ |

+ – identified, NQ – not quanified

Table S3: Methods of disinfection and preparation of disinfectant suspensions for grapevine scions disinfection before grafting in 2017.

| **Disinfection** | **Description**[1] | **Concentration (on 10 L of water)** |
|---|---|---|
| Remedier[2] | Remedier is a preventive fungicide comprising strains of antagonistic fungi, namely *Trichoderma asperellum* (strain ICC012) and *Trichoderma gamsii* (strain ICC080). These fungi, colonize the soil and roots of treated plants. They engage in competition with pathogens for resources and space, or they break down pathogen cell walls using enzymes. | 1,0 kg |
| Serenade® ASO | Preventive fungicide comprised of the in the environment naturally occurring bacterium *Bacillus amyloliquefaciens* (former *B. subtilis)* strain QST 713, which is recognized as microbial disrupters of pathogen cell membranes, capable of forming endospores and producing various antibiotics, primarily during endospore formation in low concentrations and may induce plant-mediated resistance in host plants. | 80 ml |
| BioAction ES | foliar fertilizer comprising a 3% concentration of natural e+tracts (clove, lemon juice, garlic oil, peppermint) along with copper (Cu 4.5%) and MicroSap® microcrystals (15%). According to the manufacturer, this blend can bind natural substances, penetrate through the trunks, and reach conductive tissues, enhancing the plant's immune system and fostering natural resistance to diseases. | 55 ml |
| Sodium bicarbonate | Sodium bicarbonate is a salt formed from sodium (Na) and hydrogen carbonate ($HCO3^-$). Carbonate salts are one of the newer possible alternatives in plant protection. | 50 g |
| Beltanol[3] | Beltanol is a preventive and curative fungicide and bactericide, based on a 37.37% solution of 8-hydro+yquinoline. | 70 ml |
| Beltanol + HWT | HWT: Grapevine grafts or parts of grafts (canes, scions, rootstocks) are e+posed to high temperatures (around 50 °C) in order to destroy the microorganisms, present on the wood. Most often by immersion in hot water for a few minutes to a few hours or by e+posure to high temperatures and high relative humidity for a few days. | 50 °C for 30 min + 70 ml |

[1] Descriptions are summarized according to Gačnik S. (2018). [2]prepared 24 h before use, [3]positive control

Gačnik, S. Vpliv zdravstvenega stanja cepičev ter sredstev za razkuževanje na vsebnost fenolnih spojin v kalusu cepljenk žlahtne vinske trte (*Vitis vinifera* L.) sorte “Cabernet sauvignon” [Thesis, Univerza v Ljubljani, Biotehniška fakulteta]. https://repozitorij.uni-lj.si/IzpisGradiva.php?id=102744

Table S4: HPLC and MS conditions, based on Gačnik et al. [30].

| | |
|---|---|
| | **HPLC conditions** |
| **Recorded spectra** | 200-600 nm |
| **Detector** | Diode array detector (DAD) |
| **Column** | Gemini C18 (150+4,6mm;3µm); Phenomene+ |
| **Column temperature** | 25°C |
| **Injection** | 20 µl |
| **Flow rate** | 0.6 ml/min |
| **Total run time** | 45 min |
| **Mobile phase** | A: 0.1%formic acid + 3% bi$H_2O$ + 97% acetonitrile; B: 0.1%formic acid + 3% acetonitrile + 97% bi$H_2O$ |
| | **MS conditions** |
| **Full scan (m/z)** | 115-1500 |
| **Injection volume** | 10 µl |
| **Flow rate** | 0.6 ml/min |
| **Heater and capillary temperature** | 320°C |
| **Sheat-Au+-Sweep Gas Flow Rate** | 50 – 20 – 0 arb |
| **I Spray Voltage (kV)** | 3.5 |
| **Capillary voltage (V)** | 10 |
| **Mobile phase** | A: 0.1%formic acid + 3% bi$H_2O$ + 97% acetonitrile; B: 0.1%formic acid + 3% acetonitrile + 97% bi$H_2O$ |

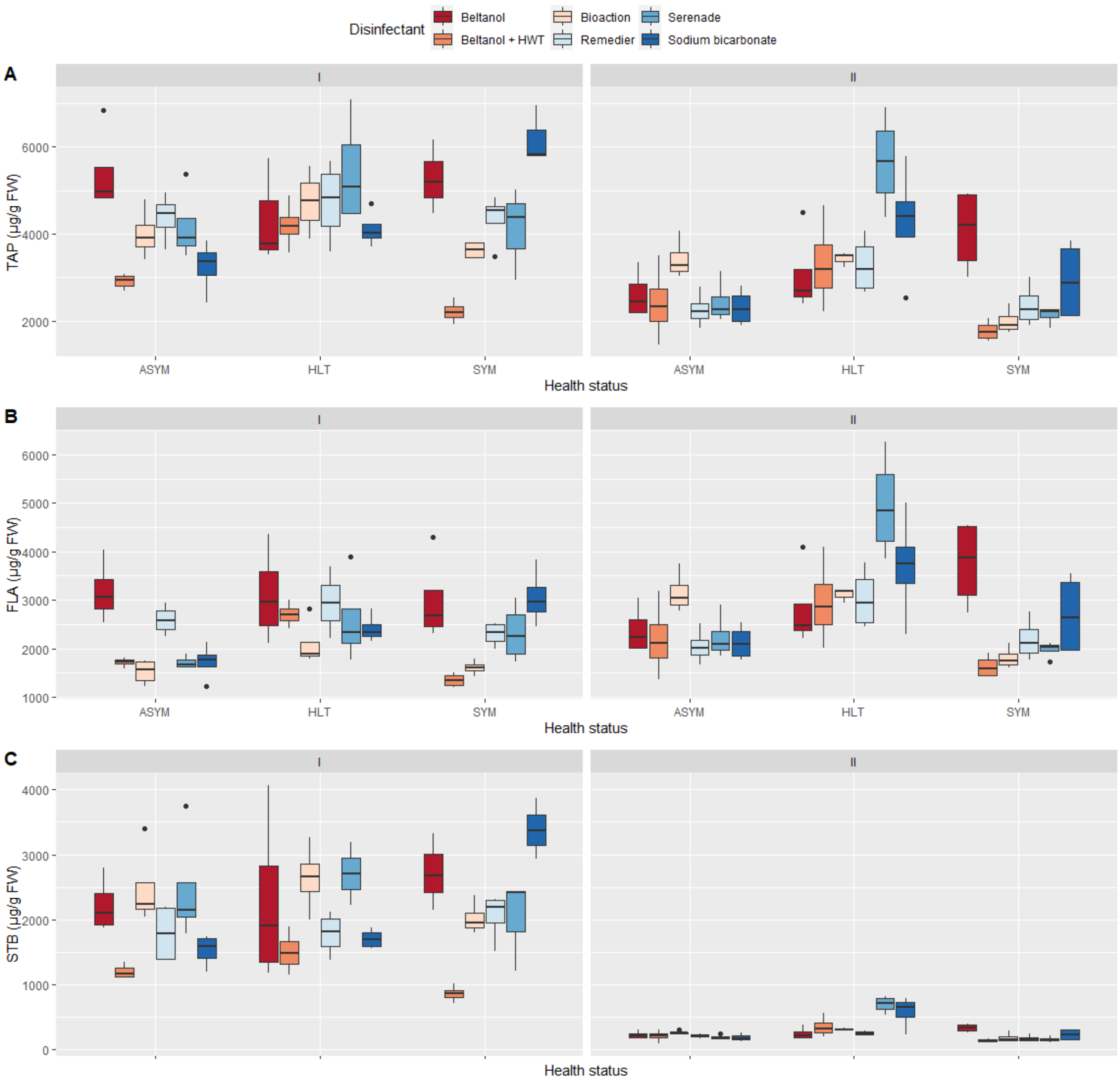


Figure S1: Content (µg/g FW) of TAP – total analysed phenolic content (A), FLA – flavanols (B), STB – stilbenes (C) in scions categorized by different health statuses (healthy: HLT, asymptomatic: ASYM, and symptomatic: SYM), disinfected prior grafting using various disinfectant methods (Beltanol, Beltanol + Hot Water Treatment (HWT), BioAction ES, Remedier, Serenade® ASO, sodium bicarbonate), sampled before grafting (I) and after graft ranking (II).

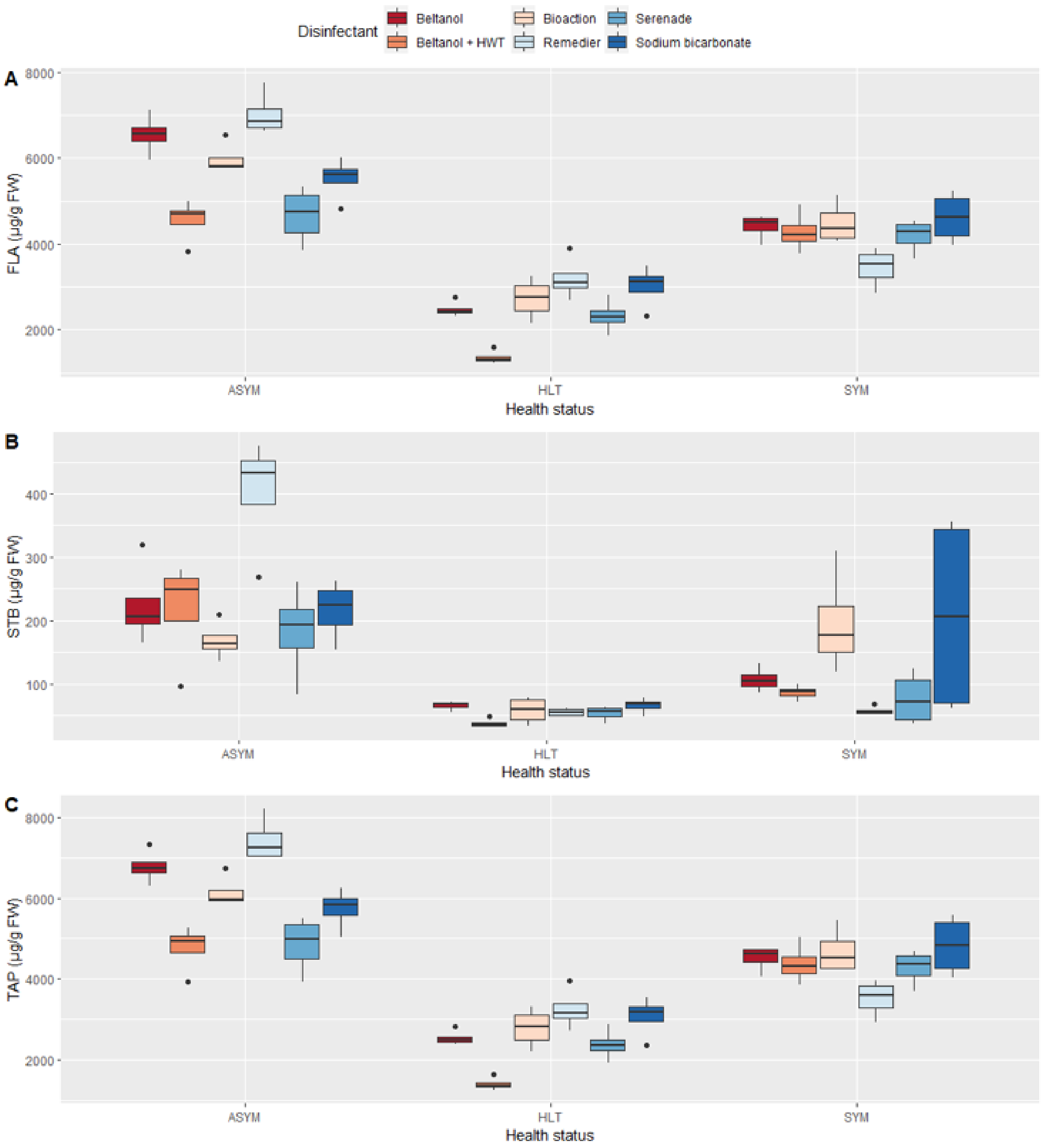


Figure S2: Content (µg/g FW) of TAP – total analysed phenolic content (A), FLA – flavanols (B), STB – stilbenes (C) in graft callus categorized by different health statuses (healthy: HLT, asymptomatic: ASYM, and symptomatic: SYM), disinfected prior grafting using various disinfectant methods (Beltanol, Beltanol + Hot Water Treatment (HWT), BioAction ES, Remedier, Serenade® ASO, sodium bicarbonate), sampled after graft ranking.

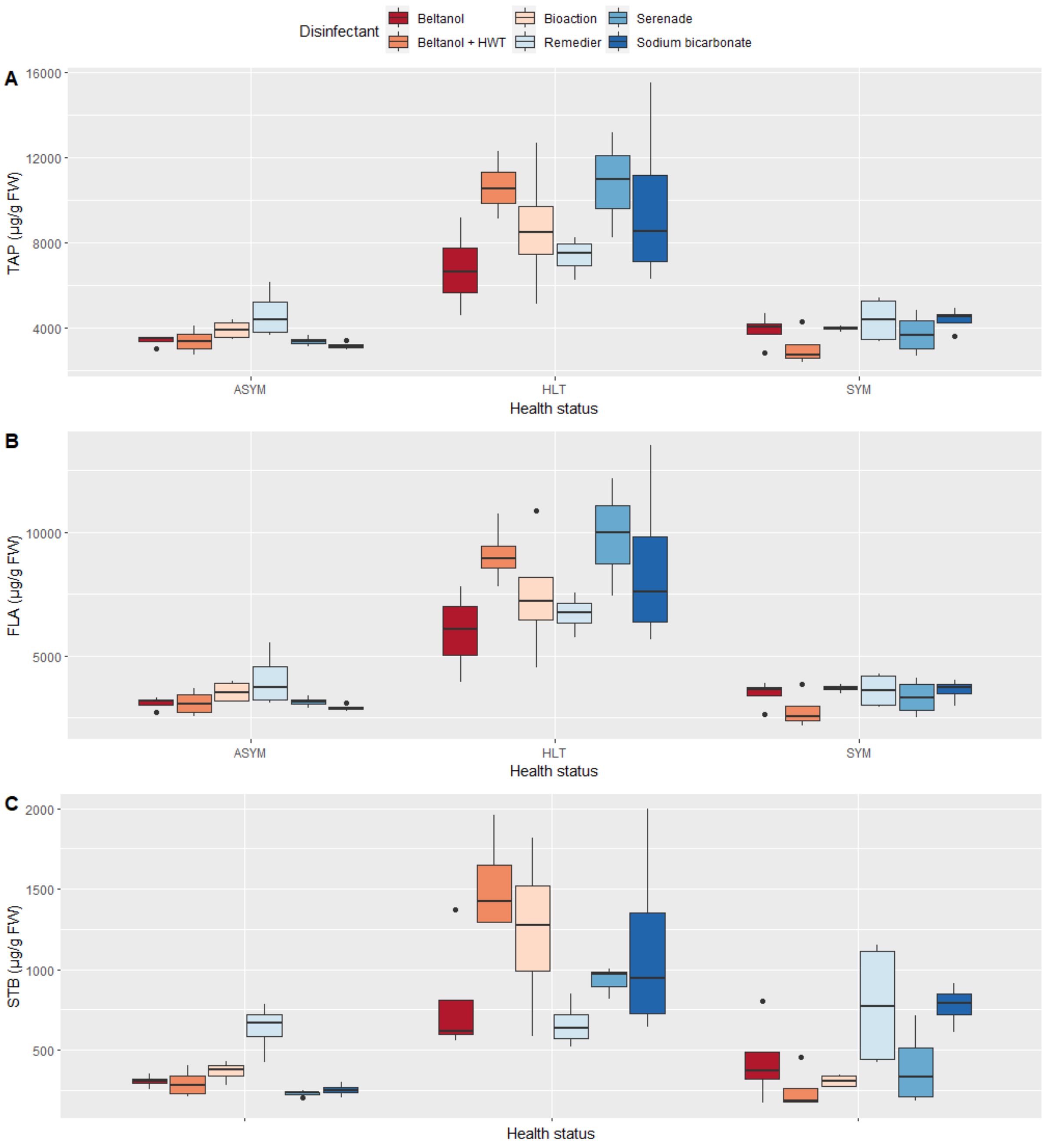


Figure S3: Content (µg/g FW) of TAP – total analysed phenolic content (A), FLA – flavanols (B), STB – stilbenes (C) in rootstock canes categorized by different health statuses (healthy: HLT, asymptomatic: ASYM, and symptomatic: SYM), disinfected prior grafting using various disinfectant methods (Beltanol, Beltanol + Hot Water Treatment (HWT), BioAction ES, Remedier, Serenade® ASO, sodium bicarbonate), sampled after graft ranking.

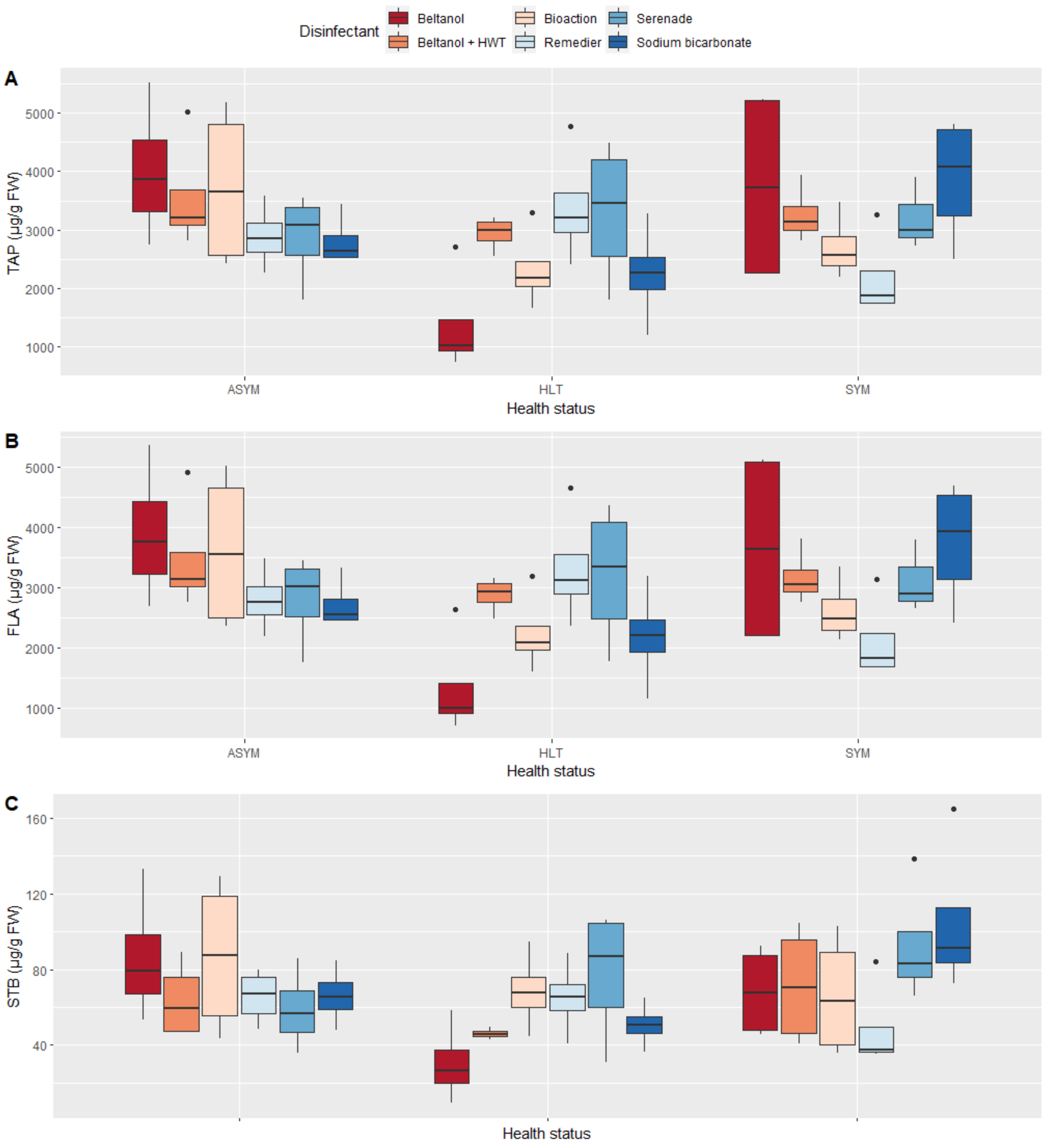


Figure S4: Content (µg/g FW) of TAP – total analysed phenolic content (A), FLA – flavanols (B), STB – stilbenes (C) in roots categorized by different health statuses (healthy: HLT, asymptomatic: ASYM, and symptomatic: SYM), disinfected prior grafting using various disinfectant methods (Beltanol, Beltanol + Hot Water Treatment (HWT), BioAction ES, Remedier, Serenade® ASO, sodium bicarbonate), sampled after graft ranking.

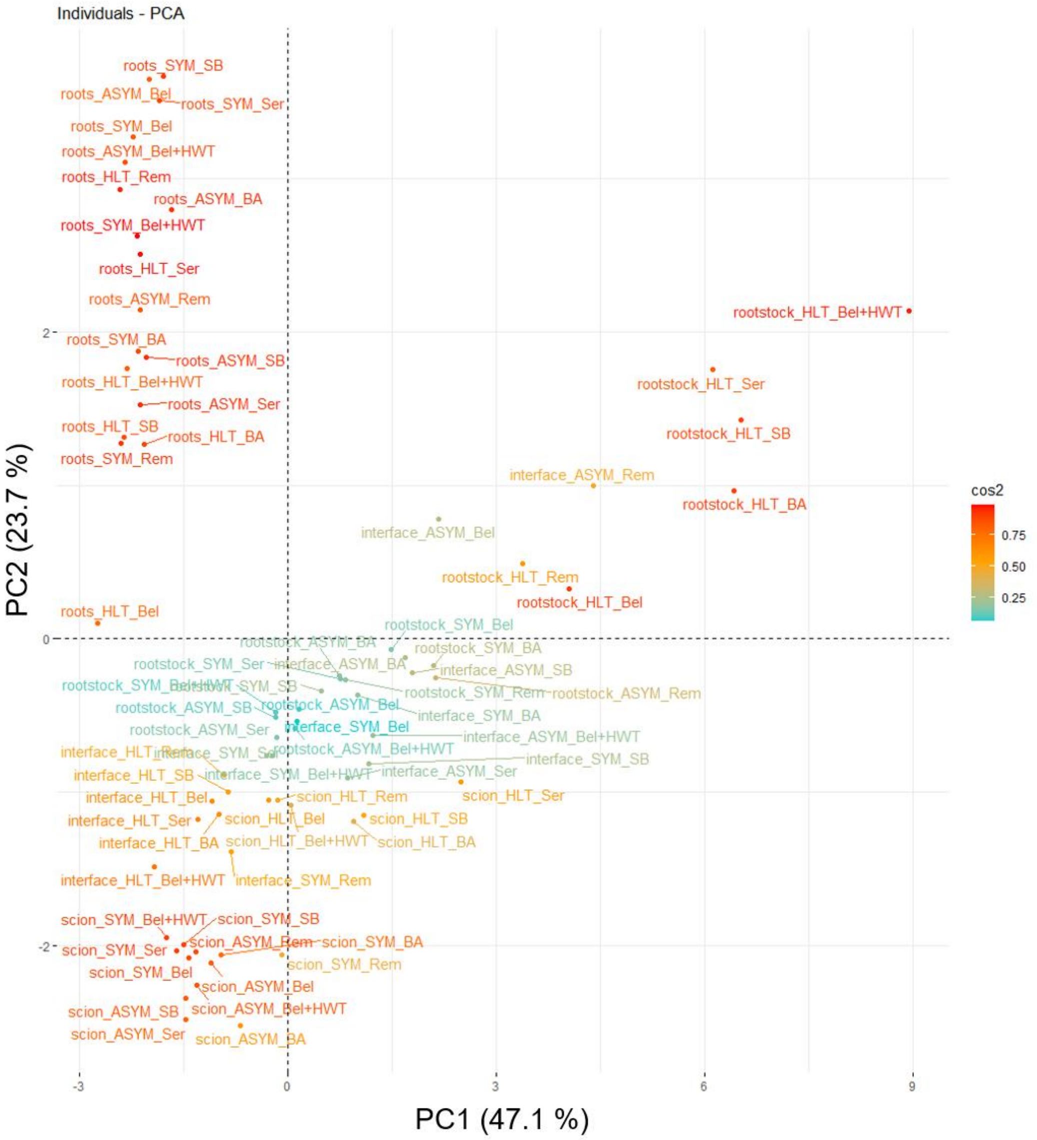


Figure S5: Principal component analysis (PCA) of metabolite content from different vine grafts part (scion, callus, rootstock canes, roots), categorized by different health statuses (healthy: HLT, asymptomatic: ASYM, and symptomatic: SYM), disinfected prior grafting using various disinfectant methods and all after graft ranking analysed phenolic compounds (n=4). PCA score plots showing the different individuals, colored by contribution strength of each combination disinfectant_health status_graft part.
